# Loss ratio of the capitation payment unit of the health-promoting entities in Colombia between 2017 and 2021: a financial–actuarial approach

**DOI:** 10.1186/s12962-023-00481-5

**Published:** 2023-10-04

**Authors:** Oscar Espinosa, Jhonathan Rodríguez, B. Piedad Urdinola, Pedro Luis Do Nascimento Silva, Alejandra Sánchez, Martha-Liliana Arias, Emiliano A. Valdez, Terence Cheng, Sara-Ellison Fisher

**Affiliations:** 1 Research Group on Economic Models and Quantitative Methods, Centro de Investigaciones para el Desarrollo, Universidad Nacional de Colombia, Bogotá, Colombia; 2 General Directorate, Departamento Administrativo Nacional de Estadística, Bogotá, Colombia; 3 Escola Nacional de Ciências Estatísticas, Rio de Janeiro, Brasil; 4 Department of Mathematics, Universidad Nacional de Colombia, Bogotá, Colombia; 5 Department of Accounting Sciences, Pontificia Universidad Javeriana, Bogotá, Colombia; 6 Department of Mathematics, University of Connecticut, Mansfield, USA; 7 Harvard T.H. Chan School of Public Health, Harvard University, Boston, USA; 8 Department of Economics, MIT, Boston, USA

**Keywords:** General System of Social Security in Health, Health-Promoting Entities, Actuarial sciences, Loss ratio, Colombia, Capitation Payment Unit

## Abstract

**Background:**

Because of a change of government, the Colombian Ministry of Health and Social Protection is in the process of presenting a structural reform for the General System of Social Security in Health (GSSSH), in order to implement a ‘preventive and predictive health model’. However, it will always be relevant to review and analyze the fiscal implications of any proposed public policy program, to protect financial sustainability and to promote the better functioning of the system in question.

**Methods:**

To contribute to this topic, we have calculated, using a financial–actuarial approach, the loss ratio for the years 2017 to 2021 for the Capitation Payment Unit (CPU) for all the Health-Promoting Entities (HPE) for both contributory and subsidized schemes. This information, derived from public reports available on the official website of the National Health Superintendency, allows us to estimate the financial burden of the institutions that guarantee access to and provision of health services and technologies in Colombia.

**Results:**

The study shows that close to half of the HPEs in Colombia (which represent 11.6 million affiliates) have CPU loss ratios of more than 100% for the year 2021, evidencing insufficient resources for the operation of health insurance.

**Conclusions:**

Finally, we propose some policy recommendations regarding the strengthening of informed decision-making to allow the healthy financial sustainability of the Colombian GSSSH.

## Introduction

Colombia’s General System of Social Security in Health (GSSSH) was created by Law 100 of 1993 [1], and defines health insurance in article 14 of Law 1122 of 2007 as:

*(…) financial risk management, health risk management, articulation of services that guarantee effective access, quality assurance in the provision of health services and the representation of the member before the provider and other actors without prejudice to the autonomy of the user* [2].

In Colombia, the companies in charge of these functions are called Health Benefit Plan Administration Entities, and, within these, the Health-Promoting Entities (HPEs) stand out. These are the companies in charge of assuming the risk transferred by the user, guaranteeing the fundamental right to health [3], complying with all the legal provisions of the respective insurance, and having as their ultimate goal the constant improvement of the health of the entire affiliated population.^1^

Currently, there are two main mechanisms for the financing of the GSSSH and the operation of the HPEs, one ex-ante^2^ and the other ex-post. The former refers to the Capitation Payment Unit (CPU), an insurance premium that is defined annually by the Ministry of Health and Social Protection on the basis of the age, sex, and region of residence of each affiliate to the HPE, through a pricing method called the loss ratio [4]. This monetary amount is the amount that the GSSSH resource administrator gives to each HPE to guarantee the provision of health services and technologies (HSTs) financed by the CPU to its entire pool of risks [5]—all GSSSH affiliates are entitled to the same set of HSTs, regardless of their HPE. The ex-post mechanism is a zero-sum adjustment according to the results of the HPE risk management for the following health conditions: chronic kidney disease, arterial hypertension, diabetes mellitus, HIV, and cancer. This is done by the High Cost Account, which is the entity responsible for the calculation and application of this resource redistribution mechanism [6].^3^

By 2022, the HST charged to the CPU represented more than 84% of the total expenditure in the GSSSH, approximately 62.4 billion Colombian pesos [7]. Likewise, it is important to highlight that 96.9% of health procedures and 93.7% of medicines approved and available for use in the country are currently fully financed with CPU resources [5].

Within the framework of social health insurance, if the CPU is conceived of as an insurance premium, its financial sufficiency is vital for the sustainability of the GSSSH in the short, medium, and long term (given the aging of the population, the increase in life expectancy, pharmacological innovations, the updating of health benefit plans, etc.). Therefore, each year the Ministry of Health and Social Protection must estimate a rate consistent with the future demand for HST that allows, in a supportive and comprehensive manner, the fundamental right to health of all affiliates to be guaranteed.

According to article 23 of Law 1438 of 2011, an HPE’s administration expenses cannot exceed 8% or 10% of the CPU for subsidized^4^ (SS) or contributory^5^ (CS) schemes, respectively. Therefore, the loss ratio, actuarially conceptualized as the cost of the set of events that have already materialized and that are covered by the insurance, cannot be less than 92% in the SS and 90% in the CS [8]. Although having a claim rate close to or greater than 100% is not adequate for financial solvency, neither is having a very low claim rate, since health insurers must invest part of their premium in the promotion of the health and prevention of illness of their members, as well as improving the efficiency and quality of care [9]. This investment fosters a positive result from two perspectives: (i) the maintenance of the health of the population of interest, and (ii) the reduction of future avoidable health conditions, especially in the medium and long term.

In recent decades at the international level, the calculation methodology and the regulatory aspects of health loss ratios for insurers have been a topic of priority interest in public supervision policy [10, 11]. The region with the greatest scientific production in this regard is North America [9, 12–16], although work has also been done for European [17], Asian [18, 19], and African [20] nations. In countries like the United States, Israel, the Netherlands, Vietnam, South Africa, Germany, Denmark, and Spain, among others, the medical loss ratio is between 49% and 92%. These values represent a wide range, since most of the health insurance in these countries is private, differing from the conceptual framework for the CPU in Colombia of social insurance.

In Colombia, the Ministry of Health and Social Protection has, for more than two decades, published an annual sufficiency report for the CPU, in which an analysis of the loss ratio is shown; however, this analysis is not carried out for all the HPEs in the GSSSH but only for those selected at the discretion of the Ministry [21].^6^ Outside the governmental sphere, only two approaches to the calculation the loss ratio have been published. In 2021, the Colombian Association of Comprehensive Medicine Companies (ACEMI, by its Spanish acronym), one of the HPE's guilds, estimated the loss ratio for both schemes, but the technical development of this report is not publicly available, and nor is its methodology, meaning that it is not transparent or replicable but is merely a ‘black box’ [22]. Subsequently, in 2023 NUMERIS estimates the loss ratio only for the contributory scheme, and although they show the methodology in a very aggregated way, they did not specify the financial sub-accounts analysed for the calculation [23].

This research article calculates the CPU loss ratio for all HPEs in the country in the contributory and subsidized schemes, between 2017 and 2021, to provide empirical evidence on the current financial conditions of HPEs in Colombia, in such a way as can nourish the debate on the options for improvement in insurance finances in the Colombian health system.

## Methodology and data

The CPU loss ratio $({CPU\_LR}_{t})$, according to the context of the GSSSH and the definition used in the actuarial sciences [24–27], is understood as a measure of the proportion of each monetary unit received as income within the concept of the CPU that is used to pay for the HST incurred under the CPU.

In formal terms, it is calculated as: 7:

1 $${CPU\_LR}_{t,i}=\left(\frac{{CPU \ Costs}_{t,i}}{{CPU \ Incomes}_{t,i}}\right)*100\%,$$

where $t$ is the year in the analysis and $i$ is the HPE of interest.

The data needed to calculate the ${CPU\_LR}_{t,i}$ of the different HPEs (both SS and CS), were extracted from the technical annex *FT001—Financial information catalog for supervisory purposes* [28], provided on the website of the National Health Superintendency, between 2017 and 2021. In Table 1 the different HPEs are presented by scheme and classification according to the International Financial Reporting Standards (IFRS) or Public Accounting Regime (PAR). The table shows that, for the different years, the HPEs analyzed in this study together cover—on average—99% of the population affiliated with the GSSSH (more than 44 million people, on average per year).

**Table 1:** Number of affiliates per HPE (expressed in hundred thousand) according to scheme and IFRS/PAR classification, years 2017 to 2021

Classification	Authorized/enabled scheme	HPE	2017	2018	2019	2020	2021
IFRS 1	Contributory	Aliansalud	2.05 (0.47%)	2.11 (0.48%)	2.25 (0.49%)	2.34 (0.51%)	2.40 (0.49%)
		Compensar	13.27 (3.03%)	14.27 (3.22%)	15.44 (3.38%)	17.34 (3.76%)	19.43 (3.96%)
		Coomeva	26.38 (6.02%)	22.68 (5.12%)	18.75 (4.1%)	15.61 (3.38%)	13.28 (2.71%)
		Sanitas	19.19 (4.38%)	23.07 (5.2%)	28.12 (6.15%)	34.82 (7.54%)	42.80 (8.73%)
		Sura	25.77 (5.88%)	29.15 (6.58%)	34.26 (7.49%)	38.98 (8.44%)	44.37 (9.05%)
	Subsidized	CCF de Nariño–Comfamiliar Nariño	1.84 (0.42%)	1.83 (0.41%)	1.85 (0.4%)	1.82 (0.39%)	1.71 (0.35%)
		CCF del Huila–Comfamiliar Huila	5.24 (1.2%)	5.73 (1.29%)	5.71 (1.25%)	5.58 (1.21%)	5.50 (1.12%)
	Mixed	Asociación Mutual Ser Empresa Solidaria de Salud–Mutual Ser	14.78 (3.37%)	15.90 (3.59%)	18.82 (4.11%)	21.10 (4.57%)	23.19 (4.73%)
IFRS 2	Contributory	Comfenalco Valle	2.41 (0.55%)	2.34 (0.53%)	2.31 (0.5%)	2.39 (0.52%)	2.57 (0.52%)
		Cruz Blanca	4.96 (1.13%)	4.33 (0.98%)	2.94 (0.64%)	0 (0%)	0 (0%)
		Famisanar	18.84 (4.3%)	20.32 (4.58%)	22.49 (4.92%)	24.75 (5.36%)	26.67 (5.44%)
		Fundación Salud Mía	0 (0%)	0.04 (0.01%)	0.17 (0.04%)	0.35 (0.08%)	0.47 (0.1%)
		Salud Total	24.69 (5.64%)	26.68 (6.02%)	29.63 (6.48%)	34.17 (7.4%)	38.88 (7.93%)
		Servicio Occidental de Salud–S.O.S	9.30 (2.12%)	9.21 (2.08%)	8.70 (1.90%)	8.31 (1.80%)	8.18 (1.67%)
	Subsidized	Asociación Barrios Unidos de Quibdó—AMBUQ	8.73 (1.99%)	8.30 (1.87%)	7.91 (1.73%)	7.71 (1.67%)	7.69 (1.57%)
		Asociación Mutual Empresa Solidaria de Salud de Nariño—EMSSANAR	19.08 (4.35%)	19.15 (4.32%)	19.07 (4.17%)	19.14 (4.15%)	19.41 (3.96%)
		Asociación Mutual la Esperanza—ASMET Salud	19.30 (4.40%)	19.02 (4.29%)	18.86 (4.12%)	18.92 (4.10%)	19.37 (3.95%)
		CCF Cajacopi Atlántico	7.82 (1.79%)	8.74 (1.97%)	10.05 (2.20%)	11.43 (2.48%)	12.82 (2.62%)
		CCF de Cartagena—Comfamiliar Cartagena	1.96 (0.45%)	1.73 (0.39%)	1.52 (0.33%)	1.38 (0.30%)	0 (0%)
		CCF de Córdoba—Comfacor	5.77 (1.32%)	5.51 (1.24%)	5.39 (1.18%)	0 (0%)	0 (0%)
		CCF de Cundinamarca—Comfacundi	1.25 (0.29%)	1.23 (0.28%)	1.66 (0.36%)	1.63 (0.35%)	0 (0%)
		CCF de la Guajira—Comfaguajira	2.07 (0.47%)	2.25 (0.51%)	2.27 (0.50%)	2.46 (0.53%)	2.57 (0.52%)
		CCF de Sucre—Comfasucre	1.14 (0.26%)	1.19 (0.27%)	1.16 (0.25%)	1.16 (0.25%)	1.18 (0.24%)
		CCF del Chocó—Comfachocó	1.61 (0.37%)	1.75 (0.39%)	1.74 (0.38%)	1.75 (0.38%)	1.78 (0.36%)
		CCF del Oriente Colombiano—Comfaoriente	1.12 (0.26%)	1.22 (0.28%)	1.37 (0.30%)	1.83 (0.40%)	1.95 (0.40%)
		Comparta	17.79 (4.06%)	17.39 (3.92%)	16.21 (3.54%)	15.37 (3.33%)	15.25 (3.11%)
		Ecoopsos	2.97 (0.68%)	3.03 (0.68%)	3.23 (0.71%)	3.27 (0.71%)	3.32 (0.68%)
		Empresa Mutual para el Desarrollo Integral de la Salud—Emdisalud	4.62 (1.05%)	4.59 (1.04%)	4.06 (0.89%)	0 (0%)	0 (0%)
	Mixed	Cooperativa de Salud y Desarrollo Integral Zona Sur Oriental de Cartagena—COOSALUD	19.61 (4.47%)	20.46 (4.62%)	22.71 (4.97%)	26.10 (5.66%)	29.62 (6.04%)
		Medimás	47.55 (10.85%)	42.67 (9.62%)	35.55 (7.77%)	25.28 (5.48%)	16.04 (3.27%)
		Nueva EPS	42.35 (9.66%)	46.15 (10.41%)	52.75 (11.53%)	67.07 (14.53%)	79.70 (16.26%)
		SaludVida	12.87 (2.94%)	12.17 (2.75%)	11.55 (2.52%)	0 (0%)	0 (0%)
PAR 6	Contributory	Empresas Públicas de Medellín—Departamento Médico	0.10 (0.02%)	0.10 (0.02%)	0.09 (0.02%)	0.09 (0.02%)	0.08 (0.02%)
PAR 7	Subsidized	Anas Wayuu (Indígena)	1.72 (0.39%)	1.85 (0.42%)	1.94 (0.42%)	2.07 (0.45%)	2.17 (0.44%)
		Asociación Indígena del Cauca—AIC (Indígena)	4.66 (1.06%)	4.78 (1.08%)	5.01 (1.09%)	5.34 (1.16%)	5.43 (1.11%)
		Asociación Indígena del Cesar y La Guajira Dusakawi (Indígena)	1.97 (0.45%)	2.08 (0.47%)	2.26 (0.49%)	2.37 (0.51%)	2.50 (0.51%)
		Capital Salud	11.62 (2.65%)	11.62 (2.62%)	11.28 (2.47%)	11.36 (2.46%)	11.69 (2.38%)
		Capresoca	1.77 (0.40%)	1.79 (0.40%)	1.75 (0.38%)	1.72 (0.37%)	1.74 (0.35%)
		Convida	5.60 (1.28%)	5.50 (1.24%)	5.38 (1.18%)	5.20 (1.13%)	5.03 (1.03%)
		Mallamás (Indígena)	3.04 (0.69%)	3.13 (0.71%)	3.21 (0.70%)	3.29 (0.71%)	3.42 (0.70%)
		Pijaos Salud (Indígena)	0.80 (0.18%)	0.84 (0.19%)	0.88 (0.19%)	0.94 (0.20%)	0.99 (0.20%)
		Savia Salud	16.71 (3.81%)	17.04 (3.84%)	16.74 (3.66%)	16.84 (3.65%)	16.62 (3.39%)
PAR 8	Contributory	Fondo de Pasivo Social de los Ferrocarriles Nacionales	0.41 (0.09%)	0.40 (0.09%)	0.38 (0.08%)	0.37 (0.08%)	0.35 (0.07%)
Percentage of all people affiliated to the GSSSH			93.79%	100%	100%	100%	100%

CCF: Family Compensation Fund; (ii) a mixed scheme refers to an HPE that is qualified/authorized to operate in both schemes (CS and SS); (iii) in parentheses the percentage of affiliates to the total number affiliated to the GSSSH for that year is shown

Next, we proceed to explain in detail the construction of the factors of Eq. (1), based on the available financial information for the HPEs disclosed by the National Health Superintendency. In the first measure, for the numerator, the costs for health care financing with the CPU for the CS and SS are derived from the financial sub-accounts displayed in Tables 2 and 3 (segmented according to IFRS and PAR group by financial catalog FT001).

**Table 2:** Financial sub-accounts of CPU costs for the contributory scheme according to IFRS or PAR group

*IFRS 1 and 2*							
Financial sub-accounts	Cost of technical reserves^a^—Settled pending payment—Health services	Cost of technical reserves—Known unliquidated—Health services	Cost of technical reserves—Pending unknown—Health services	Contracts for promotion and prevention activities	Catastrophic illnesses and high-cost illnesses	Other reserves	Cost of provision of services from own providers
Financial catalog AT FT001	61020101 61020301	61020401 61020601	61021001 61021201	61021301	61021401	61021501	61050101

*PAR 6, 7 and 8*					
Public entities before resolution 427 of 2019 of the general accounting of the nation (years 2017 to 2019)		Public entities after resolution 427 of 2019 of the general accounting of the nation (the year 2020)		Public entities after resolution 223 of 2020 of the general accounting of the nation (the year 2021)	
Capitation contracts—Contributory	561301	Capitation contracts—Contributory	561301	Promotion and prevention—Contributory	561303
Contracts by event and other modalities—Contributory	561302	Contracts by event and other modalities—Contributory	561302	Guarantee and quality system—Contributory	561304
Promotion and prevention—Contributory	561303	Promotion and prevention—Contributory	561303	High-cost disease reinsurance—Contributory	561305
Guarantee and quality system—Contributory	561304	Guarantee and quality system—Contributory	561304	Technical reserve for known unpaid health services and technologies, financed with the CPU	537201
High-cost disease reinsurance—Contributory	561305	High-cost disease reinsurance—Contributory	561305	Technical reserve for health services and technologies that have occurred and are not known	537202
Technical reserves for authorized health services	561320	Technical reserves for authorized health services	537201	Other provisions for health services and technologies	537290
Technical reserves for unknown health services occurred	561321	Technical reserves for unknown health services occurred	537202		
Other technical reserves	561323	Other provisions for health services	537290		

^a^The technical reserves can be understood as a provision that will guarantee the payment for the HST provided to the affiliates of the HPE, which is a legal requirement defined by the National Health Superintendency

**Table 3:** Financial sub-accounts of CPU costs for the subsidized scheme according to IFRS or PAR group

*IFRS 1 and 2*							
Financial subaccounts	Cost of technical reserves—Settled pending payment—Health services	Cost of technical reserves—Known unliquidated—Health services	Technical reserve cost—Unknown pending—Health services	Contracts for promotion and prevention activities	Catastrophic illnesses and high-cost illnesses	Other reserves	Cost of provision of services from own providers
Financial catalog AT FT001	61020102 61020302	61020402 61020602	61021002 61021202	61021302	61021402	61021502	61050102

*PAR 6, 7 and 8*^a^					
Public entities before Resolution 427 of 2019 of the General Accounting of the Nation (years 2017 to 2019)		Public entities after Resolution 427 of 2019 of the General Accounting of the Nation (the year 2020)		Public entities after Resolution 223 of 2020 of the General Accounting of the Nation (the year 2021)	
Capitation contracts—Subsidized	561307	Capitation contracts—Subsidized	561307	Promotion and prevention—Subsidized	561309
Contracts by event and other modalities—Subsidized	561308	Contracts by event and other modalities–Subsidized	561308	Guarantee and quality system—Subsidized	561310
Promotion and prevention—Subsidized	561309	Promotion and prevention—Subsidized	561309	High-cost disease reinsurance—Subsidized	561311
Guarantee and quality system—Subsidized	561310	Guarantee and quality system—Subsidized	561310	Technical reserve for known unpaid health services and technologies, financed with the CPU	537201
High-cost disease reinsurance—Subsidized	561311	High-cost disease reinsurance—Subsidized	561311	Technical reserve for health services and technologies that have occurred and are not known	537202
Technical reserves for authorized health services	561320	Technical reserves for authorized health services	537201	Other provisions for health services and technologies	537290
Technical reserves for unknown health services occurred	561321	Technical reserves for unknown health services occurred	537202	Other expenses for the administration of social security in health	561390
Other technical reserves	561323	Other provisions for health services	537290		
Other expenses for the administration of social security in health	561390	Other expenses for the administration of social security in health	561390		

^a^In public catalogs (AT-FT001-06, AT-FT001-07, AT-FT001-08) it is not possible to determine, in the sub-accounts related to technical reserves, whether those are from the SS or the CS, and therefore this should be considered in accordance with the authorized/enabled regime of the study HPE

We consider that the release of reserves,^8^ although it is part of the income account (non-operational), should be read as a minor cost since it directly impacts technical reserves. Therefore, the sum of the costs must be subtracted from the monetary values consigned to the financial sub-accounts related to the release of technical reserves, so that for the HPEs in IFRS 1 and 2 the following three financial sub-accounts must be taken: (i) 410204 (‘Release of technical reserves—Pending and known obligations’), (ii) 410205 (‘Release of technical reserves—Unknown outstanding obligations’) and (iii) 410206 (‘Release of technical reserves—Other reserves’); while for the HPEs in IFRS 6, 7 and 8 this corresponds to the financial sub-account 435508 (‘Release of technical reserves’). These financial items should theoretically be used when reserves from previous periods are released instead but, in practice, they have been used in the current release, so that fact should be reflected as a lower cost and not as income.

Secondly, the denominator is the CPU income for the HPE resulting from the sum of the financial sub-accounts presented in Table 4, according to its IFRS or PAR group, and according to the financial catalog AT FT001.

**Table 4:** CPU income financial sub-accounts for the contributory and subsidized schemes according to IFRS or PAR group

Contributory scheme					
Financial catalog AT FT001	Financial sub-accounts				
	Capitation Payment Unit–CPU	Additional CPU^a^	Payment unit for promotion and prevention activities	Moderator fees	Copays
IFRS 1 and 2	41020101	410202	410203	41020801	41020901
PAR 6, 7 and 8	431101	431102	431122	431103	431104

Subsidized scheme		
Financial catalog AT FT001	Financial sub-accounts	
	Capitation Payment Unit–CPU	Copays
IFRS 1 and 2	41020102	41020902
PAR 6, 7 and 8	431106	431107

^a^This sub-account corresponds to an additional premium that is delivered to the HPE by affiliates residing in certain municipalities due to geographic dispersion and low population density, in order to cover cost overruns in health care

For the SS and CS, the financial sub-accounts related to disabilities, complementary care plans, and maximum budgets were excluded from the analysis, since these are not charged to the CPU.

On the other hand, the estimation takes into account what is referred to as mobility between schemes. This aspect is important for calculating the loss ratio due to the HPE in the GSSSH, because it impacts a particular financial fact: an HPE authorized/enabled to operate in the CS may have members of the SS, and therefore resources associated with this latter health scheme [29]. Likewise, an SS HPE may have CS affiliates, with their respective financial resources.

The foregoing follows the provisions of article 2.1.1.3 of Decree 780 of 2016, which defines ‘*(…) the change of belonging to a scheme within the same HPE for affiliates in the General System of Social Security in Health focused on levels I and II of the SISBEN [Identification System for Potential Beneficiaries of Social Programs] and some special populations*’ [30]. This mechanism allows continuity of insurance for vulnerable people, so that if, for example, a person belonging to the CS lost their ability to pay, they would move to the SS, but would continue to be affiliated with the same HPE.

Therefore, Eq. (1) is redefined as follows:

2 $$CPU\_LR_{{t,i}} = \left( {\frac{{\begin{array}{*{20}c} {CPU\,~Costs\,~from\,~Affiliates~\,CS_{{t,i}} ~ + CPU\,~Costs~\,from\,~Affiliates\,~SS_{{t,i}} } \\ { -\, Release~CPU~Reservations_{{t,i}} } \\ \end{array} }}{{CPU~\,Incomes~\,from~\,Affiliates\,~CS_{{t,i}} ~\, + \,~CPU~\,Incomes\,~from\,~Affiliates~\,SS_{{t,i}} }}} \right)*100\% .$$

In this way, it is possible to consolidate the financial information^9^ necessary to properly calculate the ${CPU\_LR}_{t,i}.$

## Results

For the HPEs in the IFRS 1 and IFRS 2 categories, more than 80% of the costs are associated with ‘Cost of technical reserves—Settled pending payment—Health services’ (71.41% annual average) and ‘Catastrophic illnesses and high-cost illnesses’ (9.50% annual average), which have been increasing during the analysis period. In monetary terms, the two sub-accounts with the highest growth rates between 2017 and 2021 are ‘Technical reserve cost—Unknown pending—Health services’ and ‘Cost of technical reserves—Known unliquidated—Health services’, which have increased^10^ by 280.05% and 201.13%, respectively. The ‘Other reserves’ sub-account is, from 2020, one of the ones with the lowest participation in costs and the only one that has decreased, going from $2.06 trillion in 2017 to $240 billion Colombian pesos in 2021.

On the other hand, for the HPEs in the PAR 6, PAR 7, and PAR 8 categories, between 2017 and 2020 close to 80% of the costs were concentrated in ‘Contracts per event and other modalities’ (55.90% annual average) and ‘Capitation contracts’ (32.19% annual average); however, in 2021 there was a change, with ‘Other expenses for the administration of social security in health’ being the highest cost, going from a share of 7.48% of total costs in 2017 to 72.10% in 2021. The account with the greatest variation is the ‘Guarantee and quality system’, which increased its monetary amount by more than 50 times in the analysis period, going from $4.86 billion in 2017 to $363.33 billion Colombian pesos in 2021.

Regarding the income related to the CPU, both for the HPEs in the IFRS 1 and 2 categories and those in the PAR 6, 7, and 8 categories, about 97% of the income corresponds to the ‘Capitation Payment Unit—CPU’, which is foreseeable given the logic of this financial item. Regarding the release of reserves, the results identify that this is done only by HPEs in the IFRS 1 and IFRS 2 categories, with the sub-account ‘Release technical reserves—Pending and known obligations’ being the one with the highest share of the total releases (85.42% annual average). These releases represent, on average, 2.73% of the costs.

**Fig. 1:**
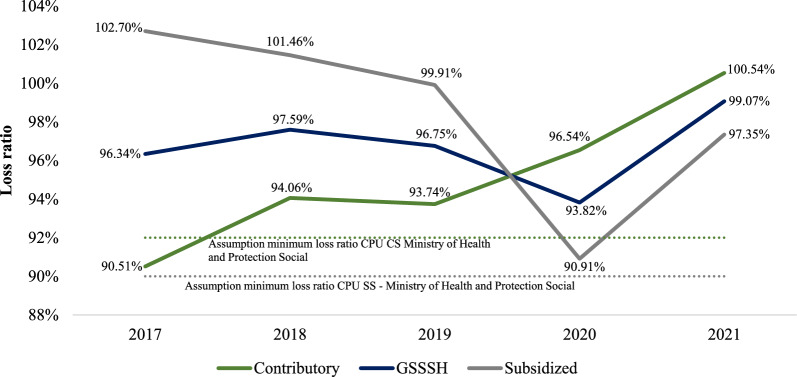
CPU loss ratio by scheme, 2017–2021. CPU Capitation Payment Unit, CS Contributory scheme, SS Subsidized scheme, GSSSH General System of Social Security in Health

The GSSSH of Colombia has a decrease in its loss ratio between 2018 and 2020, going from 97.59% to 93.82%; however, in 2021 there is an increase of 5.25 percentage points (p.p.), with the ratio reaching 99.07%. When estimating the aggregate CPU^11^ loss ratio by the scheme, the SS shows a decrease of 11.79 p.p., going from 102.70% in 2017 to 90.91% in 2020; however, this trend is reversed in 2021, when there is an increase of 6.44 p.p. to a loss ratio of 97.35%. For its part, the CPU claims ratio of the CS, in general, has increased over the period, from 90.51% in 2017 to 100.54% in 2021^12^, which implies an increase of 10.03 p.p. It is noteworthy that, except for the CS in 2017, the $CPU\_LR$ estimated here has been higher than the minimum value regulated by the Ministry of Health and Social Protection (Fig. 1).

**Fig. 2:**
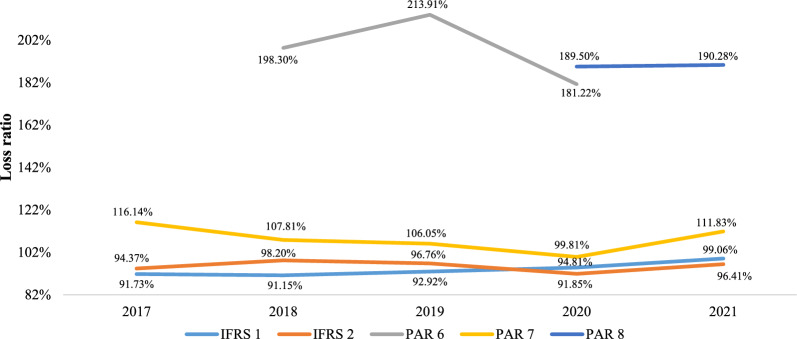
CPU loss ratio by IFRS and PAR category, 2017–2021. IFRS International Financial Reporting Standards, PAR Public Accounting Regime

According to the type of accounting that is carried out, and taking into account the nature of the HPEs, it can be observed that the loss ratio of the HPEs of the PAR 6, PAR 7, and PAR 8 categories is, throughout the analysis period, superior to the rest, and in some cases, is almost double the index for the HPEs of the IFRS 1 and IFRS 2 categories (Fig. 2). This difference is because the HPEs that belong to the PAR 6 and PAR 8 categories, *EPM Empresa Pública de Medellín* and *Fondo de Pasivo Social de los Ferrocarriles Nacionales*, respectively, are institutions whose corporate purpose is not health insurance (they are so-called adapted health entities), so that only their own workers and beneficiaries are affiliates, they have a regressive population pyramid (a high proportion of people in old age), and they have high capitalization and operation costs and financial insolvency [31].

Table 5 shows the calculation of the ${CPU\_LR}_{t,i}$ for each entity for the different years of study (2017–2021), from which one can observe that, on average, the loss ratio of 44.19% of the insurance entities analyzed exceed 100%. Given the previously mentioned characteristics of *EPM Empresa Pública de Medellín* and *Fondo de Pasivo Social de los Ferrocarriles Nacionales*, it is evident that these two companies have the highest loss ratios. It should be noted that all the companies whose affiliates are original indigenous populations had, at least one moment of the analysis period, indices above 100%. Likewise, firms whose loss ratio has presented greater variations between 2017 and 2021 are authorized/enabled to operate in the SS (*Emdisalud*, *Savia Salud, Comfamiliar Nariño*, *Pijaos Salud* and *Comfacundi*).

**Table 5:**
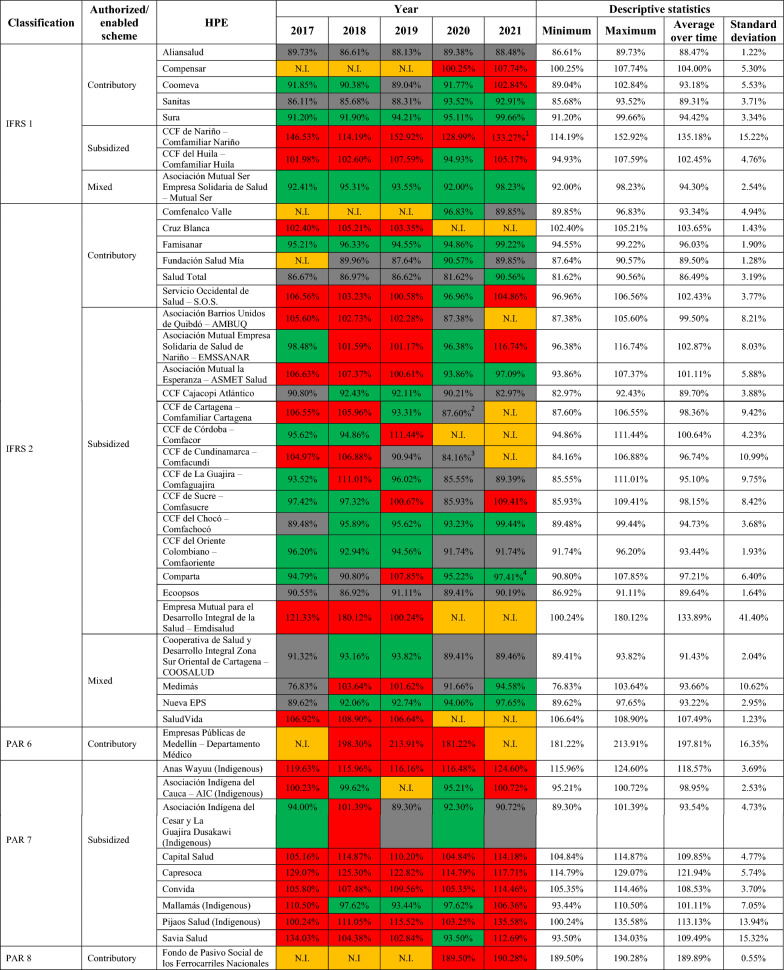
CPU loss ratio (%) by HPE (including mobility), 2017–2021

Red: ${CPU\_LR}_{t,i}$ higher than 100%; green: ${CPU\_LR}_{t,i}$ between the legal minimum according to the scheme (90% for HPE operating in the CS and 92% for HPE operating in the SS and mixed) and 100%; grey: ${CPU\_LR}_{t,i}$ lower than the imposed legal minimum; orange: no information (N.I.) – either because the HPE’s information report is inconsistent or because it is in liquidation

^1^Available information as of September 2021

^2^Available information as of September 2020

^3^Available information as of September 2020

^4^Available information as of March 2021

## Discussion and conclusions

Every health system must have, as its most important premise, the maximization and improvement of the health results of the general population, and thus must be aiming for the financial sustainability of the different actors (patients, government, providers, insurers, pharmaceutical companies, etc.) in a way that allows a sustainable, relevant, and orderly performance within clear institutional rules.

This research article estimated the loss ratios, considering the CPU concept, for more than 40 HPEs of the GSSSH of Colombia in the five years between 2017 and 2021. The results obtained in this investigation show that in the most recent year analyzed, a large proportion of the HPEs (with 66.60% of the affiliated population as of 2021) had a CPU loss ratio greater than 95%, leaving little room for administrative expenses and, in some periods, income for their proper operation that was insufficient to guarantee the fundamental right to health (as was the case for the CS added for the year 2021). In addition, it was found that the COVID-19 pandemic had a downward impact on the behavior of the index for SS in 2020, due to the dispersion and natural physical distance in scattered rural areas [32].

On the other hand, several HPEs exhibit CPU loss ratios greater than 100%, with the maximum values being obtained in the SS. As the two schemes have populations with completely different risk profiles [33], there is an urgent need to use this information for an actuarial estimation of the CPU of the SS (to which more than 24 million people belong), since the Ministry of Health and Social Protection has generally made decisions regarding its growth based on whatever happens with the behavior of the CS, as was the case in 2023.

The heterogeneity of the loss ratios may reflect divergences in the efficiency of risk management due to socio-demographic differences in their population groups, different contracting models with their network of health service providers, and inequities in the structure and availability in the supply of health services, among other systematic factors. That is why, for any new reform to the GSSSH that may be proposed, it will be essential to consider the future fiscal impact of the new health care models, as well as strengthening the supervision systems, in order to determine the effects of spending on the population health outcomes. The National Health Superintendency must play a fundamental and active role in monitoring HPE risks, a mission that up to now it has not been able to fulfill completely [34].

Likewise, it will be key that, in the debate on the future reform of the GSSSH, such important issues are addressed as the maximum value of the margin of administration by the HPE (depending on different parameters, such as size, among other characteristics), the maximum limit of 30% of the health expense of an HPE of the CS in its network of providers (Art. 15 of Law 1122 of 2007) [2], and the obligation for an SS HPE to contract a minimum of 60% of its health expenses with public providers (Art. 16 of Law 1122 of 2007) [2], among others. All these thresholds are established in the legislation of the health sector in Colombia, and impact in one way or another the comprehensive risk management of the HPE and therefore the financial sustainability of the GSSSH, and they will have to be reformulated based on scientific studies and evidence from the real contexts of these entities.

The findings set out above require caution in their reading, since they approximate a methodological proposal that provides empirical evidence on HPE loss ratios but are not the only way in which these could be calculated. The interpretation of these results must be framed under the limitations given by Robinson [35] in calculating loss ratios in health, which include: (i) these ratios are subject to somewhat arbitrary accounting conventions 13; (ii) these ratios are not the only valid measure of financial performance; (iii) low or high values are not necessarily good or bad, since the conclusion depends on the nature of the insurer and the context of the health system, and (iv) the loss ratio alone does not indicate the quality of health care.

In this way, for example, the fact that the financial policies for the accrual of health spending by CPU may vary from one HPE to another in Colombia should be taken into account. In future, the Ministry of Health and Social Protection and the National Health Superintendency could strengthen the calculation of this index through joint work with the HPEs, allowing the estimation of technical reserves and their impact on the calculated loss ratio to be homologated in a certain way. Likewise, the Ministry of Health and Social Protection should work to develop better methodological proposals for CPU pricing (for example, including health conditions or improving the accuracy of demographic forecasts) that allow a minimum financial sufficiency and avoid indebtedness and an unsustainable increase in portfolio for the health insurers. Finally, as a future line of research, the authors consider that the minimum solvency requirements required for HPEs should be studied; this is a path recently started by National Health Superintendency, but there is a long way to the horizon.

## Data Availability

The datasets and programming codes used, generated, and analyzed during the current study are available in https://github.com/Jonavier/Loss_Ratio_CPU.

## Abbreviations

CPU: Capitation Payment Unit
CS: Contributory scheme
GSSSH: General System of Social Security in Health
HPE: Health-Promoting Entities
IFRS: International Financial Reporting Standards
PAR: Public Accounting Regime
SS: Subsidized scheme

## Footnotes

1 The results obtained in this research are the sole responsibility of the authors and not of the institutions with which they are linked.

2 There is another ex-ante mechanism called maximum budgets, through which health services and technologies not covered by the CPU are financed. However, in terms of health expenses for 2022, these represented less than 5% of the total GSSSH.

3 The transfers received by the High Cost Account from the HPEs originate in the CPU, and the HPEs that benefit from these resources record these entries as operating income [29].

4 The subsidized scheme is the mechanism through which the most socio-economically vulnerable members of the population have access to health services based on subsidies offered by the state [36].

5 The contributory scheme is the mechanism through which individuals access the GSSSH through the payment of an individual and family contribution, or a previous economic contribution financed directly by the affiliate or by agreement between the latter and their employer [37].

6 For more details, see the website of the Ministry of Health and Social Protection, where it is possible to review the different studies developed on HPE spending, under the subsection *‘Estudio de Suficiencia’*, since 2006.

7 This, framed from an approach of incurred claims, technically determines the sum of paid claims plus the constitution of reserves.

8 In public catalogs it is not possible to determine, in the sub-accounts related to release of reserves, whether those are from the SS or the CS, and therefore this should be considered in accordance with the authorized/enabled regime of the study HPE. In the case of mixed HPEs, the distribution of the reserve release amount for each of the regimes is based on the distribution of the number of affiliates in each regime for the HPE of interest.

9 The data used were those reported by the HPE in the financial catalog FT001, and no adjustment was made to homogenize the applied measurement bases. The authors consider this topic an important opportunity for improvement that should be addressed by the Ministry of Health and Social Protection and the National Health Superintendency in relation to accounting standardization throughout the health insurance sector in the country.

10 Variations are calculated with deflated monetary amounts at 2021 prices.

11 For this case, the sub-accounts belonging to the SS (Table 3) of all HPEs are added, regardless of whether or not the entities are authorized/enabled to operate in the SS. The same reasoning applies in the case of the aggregate CPU loss ratio of the CS.

12 This provides evidence that, on average and according to the figures reported by the National Health Superintendency, the costs for the HPEs, once the release of reserves is excluded, are growing more quickly than their income with regard to the set of HSTs financed by the CPU.

13 In recent years, some authors have studied the impact of this type of potential bias in financial reporting, for more details see Fang et al. and Samuels et al. [38, 39].
